# Effects of nurse-led shared decision-making support based on a decision aid on decisional conflict in patients with pulmonary nodules: a randomized controlled trial

**DOI:** 10.1186/s12912-026-05040-w

**Published:** 2026-07-29

**Authors:** Jingmin Yuan, Haiyan Xu, Yan Sun, Mingwei Chen, Hui Ren

**Affiliations:** 1 Health Science Center, Yangtze University, Jingzhou, China; 2 Department of Pulmonary and Critical Care Medicine, The First Affiliated Hospital of Xi’an Jiaotong University, Xi’an, China

**Keywords:** Pulmonary nodules, Decisional conflict, Shared decision-making, Nurse-led

## Abstract

**Objective:**

To evaluate the effect of nurse-led shared decision-making support based on a decision aid on decisional conflict among patients with pulmonary nodules, and to explore its effects on psychological distress, satisfaction with participation in decision-making, and pulmonary nodule-related knowledge.

**Methods:**

The intervention group received nurse-led shared decision-making support using a decision aid in addition to physician treatment recommendations, whereas the control group received standard care. Decisional conflict, psychological distress, satisfaction with participation in decision-making, and pulmonary nodule-related knowledge were assessed immediately after the intervention (T1), at 3 months (T2), and at 6 months (T3). Causal-steps mediation analysis was conducted to examine the mediating effects of pulmonary nodule-related knowledge and satisfaction with participation in decision-making. Generalized estimating equations were used to analyze longitudinal differences between groups.

**Results:**

A total of 98 patients were enrolled, including 47 in the intervention group and 51 in the control group. The intervention group had a significantly lower decisional conflict score than the control group after the intervention (22.87 ± 17.49 vs. 48.02 ± 19.93; *t* = 6.58, *p* < 0.001). The intervention group also had significantly higher scores for satisfaction with participation in decision-making and pulmonary nodule-related knowledge than the control group (both *p* < 0.001). Mediation analysis showed that shared decision-making support significantly reduced decisional conflict (B = −25.05, 95% CI −32.74 to −17.36), and this effect was mediated by improvements in pulmonary nodule-related knowledge and satisfaction with participation in decision-making. Longitudinal analysis showed that psychological distress decreased more rapidly in the intervention group during follow-up, with significant between-group differences at T1 and T2 (*p* < 0.001).

**Conclusion:**

Nurse-led shared decision-making support based on a decision aid reduced decisional conflict among patients with pulmonary nodules when integrated into routine clinical care. This effect was mainly mediated by improvements in pulmonary nodule-related knowledge and satisfaction with participation in decision-making. The intervention also showed beneficial effects in reducing psychological distress during follow-up.

**Trial Registration:**

The study was registered in the Chinese Clinical Trial Registry on March 20, 2023(ChiCTR2300069553, retrospectively registered).

**Supplementary Information:**

The online version contains supplementary material available at https://doi.org/10.1186/s12912-026-05040-w.

## What is already known

• The detection of pulmonary nodules has increased substantially, and their management often involves multiple diagnostic options without a single optimal strategy.
• Uncertainty regarding malignancy frequently leads to decisional conflict and psychological distress among patients with pulmonary nodules.
• Although shared decision-making is recommended for preference-sensitive decisions, evidence on nurse-led shared decision-making interventions in pulmonary nodule management remains limited.

**Supplementary Information:**

The online version contains supplementary material available at https://doi.org/10.1186/s12912-026-05040-w.

## What this paper adds

• Nurse-led shared decision-making support using a decision aid significantly reduces decisional conflict in patients with pulmonary nodules.
• The beneficial effect of shared decision-making support on decisional conflict is primarily mediated by improved disease-related knowledge and satisfaction with decision participation.
• Shared decision-making support is associated with reduced psychological distress and improved adherence to planned pulmonary nodule management.

**Supplementary Information:**

The online version contains supplementary material available at https://doi.org/10.1186/s12912-026-05040-w.

## Background

Pulmonary nodules are radiographic opacities within the lung parenchyma with a maximum diameter of 3 cm or less. With the widespread use of low-dose computed tomography (LDCT) for lung cancer screening, the detection of pulmonary nodules has increased substantially. In Chinese health examination populations, pulmonary nodules are detected in up to 25% of individuals, whereas only 6.34% are pathologically confirmed as malignant tumours [1]. The risk of malignancy varies considerably according to radiographic features, such as spiculation and lobulation, ranging from 0.3% to 24.3% [2]. Accurately distinguishing benign from malignant nodules remains clinically challenging, and there is no single optimal management strategy for all patients. Common management options include serial CT surveillance at 3–6-month intervals, computed tomography-guided percutaneous transthoracic needle biopsy, bronchoscopic diagnostic procedures, and thoracoscopic surgical resection. These options differ in their risk–benefit profiles and involve varying degrees of uncertainty. For example, although CT surveillance is non-invasive, it may delay timely intervention for malignant nodules. Conversely, biopsy and surgical resection may provide a definitive diagnosis in the short term but carry risks of procedure-related complications, and surgical resection may also lead to overtreatment [3].

From the patient perspective, however, there is often a strong desire for a clear and definitive management plan [4]. The mismatch between patients’ expectations and the inherent uncertainty of available options may lead to decisional conflict or even decisional paralysis. Decisional conflict refers to uncertainty about which course of action to take when choices involve risks or uncertain outcomes [5]. Patients experiencing decisional conflict must weigh the potential benefits and risks of each option and consider the consequences of choosing or declining an intervention. In current clinical practice, pulmonary nodule management decisions are often based on paternalistic or informed decision-making models, in which physicians determine and communicate the option they consider most appropriate according to clinical characteristics and professional expertise [6]. However, our previous research showed that such a “full delegation” model does not alleviate patients’ negative emotions or decisional conflict [7]. Instead, most patients with pulmonary nodules are willing to participate in decision-making, and greater participation is associated with lower decisional conflict. Decisional conflict may also contribute to a negative cycle, as it is positively associated with psychological distress and may increase psychosocial and emotional burden [8].

Differences in decision-making attitudes and preferences between physicians and patients are common. During clinical communication, physicians and patients may hold different views about whether cancer risk should be discussed explicitly [9]. To avoid provoking anxiety, clinicians may use indirect illness metaphors rather than pathological terminology, provide limited information, or avoid individualized malignancy risk assessments. In contrast, patients often wish to obtain clear information about nodule characteristics and cancer risk, particularly when radiology reports or consultations are ambiguous or inconclusive. Vague or evasive communication may therefore heighten anxiety and lead patients to overestimate the malignant potential of pulmonary nodules. Consequently, patients may prefer more aggressive management strategies during decision-making in order to obtain diagnostic certainty and maximize perceived health benefits [10, 11]

Given the absence of a universally optimal management option, shared decision-making has become an important approach in pulmonary nodule management [12]. Shared decision-making is a collaborative process in which healthcare professionals and patients exchange information, clarify patients’ values and preferences, and jointly consider the risks and benefits of available options when no single best option exists [13]. However, implementing shared decision-making in pulmonary nodule management is challenging because of limited consultation time, insufficient communication skills, and information asymmetry. Decision aids may help address these barriers by presenting complex medical information in a structured and understandable format, facilitating information exchange, and improving the efficiency of clinical communication. Therefore, we conducted a randomized controlled trial among patients with pulmonary nodules detected through health examinations to evaluate the effect of nurse-led shared decision-making support based on a decision aid on decisional conflict and to assess the feasibility and effectiveness of implementing shared decision-making in this context.

## Methods

### Study design

This randomized controlled trial was conducted between July and October 2023 in the respiratory outpatient clinic of a tertiary teaching hospital in Xi’an, China. The study was reported in accordance with the CONSORT guidelines. Participants in the intervention group received nurse-led shared decision-making support based on a decision aid in addition to routine care, whereas participants in the control group received routine care alone. Outcomes were assessed immediately after the intervention (T1), at 3 months (T2), and at 6 months (T3). Decisional conflict was the primary outcome. Secondary outcomes included psychological distress, satisfaction with participation in decision-making, pulmonary nodule-related knowledge, and treatment adherence.

The study protocol was approved by the Institutional Review Board of the First Affiliated Hospital of Xi’an Jiaotong University (Approval No. XJTU1AF2022LSK-276). The study was conducted in accordance with relevant ethical principles, and all participants provided written informed consent before enrolment. Participant confidentiality was strictly maintained, and all collected data were used only for research purposes. The trial was registered in the Chinese Clinical Trial Registry on March 20, 2023 (ChiCTR2300069553).

### Participants

Participants were eligible if they met the following criteria: (1) aged 18 years or older; (2) had a newly detected, non-calcified pulmonary nodule measuring 5–30 mm in diameter on routine chest CT during a health examination; (3) had received at least two alternative management recommendations from the treating physician; (4) had not made a final management decision at enrolment; and (5) provided written informed consent to participate in the study.

Participants were excluded if they had a previous diagnosis of malignancy; other medical conditions that could influence pulmonary nodule management decisions, such as conditions requiring changes in the frequency of chest CT follow-up; communication or cognitive impairment; planned participation in a hospital-based multidisciplinary team discussion for pulmonary nodules; or concurrent participation in another clinical trial.

### Randomization and blinding

Eligible participants were recruited by consulting respiratory physicians from the respiratory outpatient clinic of a tertiary hospital in Xi’an, China. After eligibility screening, written informed consent, and baseline assessment, participants were randomly assigned in a 1:1 ratio to either the intervention group or the control group. Randomization was performed by an independent researcher who was not involved in participant recruitment, intervention delivery, outcome assessment, or statistical analysis. A mobile random number generator was used to generate either “1” or “2”. Participants assigned “1” were allocated to the intervention group, whereas those assigned “2” were allocated to the control group.

To reduce the risk of selection bias, the allocation sequence was generated only after participant enrolment and completion of baseline assessment. The respiratory physicians responsible for recruitment and enrolment had no access to the randomization process before participants were enrolled. After allocation, group assignment was communicated to the intervention team to enable delivery of the assigned intervention.

Because of the nature of the intervention, blinding of participants and intervention providers was not feasible. The intervention involved nurse-led shared decision-making support using a decision aid and included explicit communication, interaction, and structured consultation procedures. Therefore, participants in the intervention group were aware that they had received additional decision-support consultations. Several measures were taken to minimize potential bias. Follow-up outcome data at T2 and T3, including psychological distress, pulmonary nodule-related knowledge, and treatment adherence, were collected by researchers who were not involved in intervention delivery and were blinded to group allocation where feasible. Participants were also instructed not to disclose their group assignment during follow-up assessments. In addition, statistical analyses were conducted by a statistician who remained blinded to group allocation, with group labels coded before analysis.

### Interventions

#### Development and pilot testing

The pulmonary nodule management decision aid and the accompanying shared decision-making support programme were developed by a multidisciplinary team comprising respiratory physicians, a thoracic surgeon, a senior respiratory nurse, and doctoral nursing researchers. Development was guided by patient-centred care theory and the three-step shared decision-making model, and was informed by a previous systematic review [14], relevant clinical guidelines, and expert consensus statements.

The decision aid was an illustrated, paper-based tool designed for face-to-face use. It provided information on pulmonary nodules and compared scheduled CT surveillance, biopsy, and surgical resection in terms of procedures, potential benefits, risks, limitations, and diagnostic performance. It also supported clarification of patients’ values and preferences and documented the final management decision. All information was presented in plain, balanced, and non-directive language.

The decision aid and intervention programme were refined through two rounds of Delphi expert consultation. Before the formal trial, the procedures were pilot tested in 20 eligible patients, with 10 participants in each group, to assess feasibility and refine recruitment, physician-to-nurse referral, consultation delivery, intervention duration, and outcome assessment. Pilot participants were not included in the final analysis. Based on the pilot findings, recruitment and intervention were conducted after completion of all clinically required examinations, and the referral pathway, consultation setting, intervention sequence, duration, and core content were standardized. Detailed pilot results are presented in Table S1. The full intervention programme and decision aid are provided in Table S2 and Additional file 1, respectively.

All intervention providers received standardized training in shared decision-making, pulmonary nodule management, communication skills, and use of the decision aid. Training included completion of the Ottawa Decision Support Tutorial [15], review of relevant guidelines and expert consensus statements, and clinical practice in the respiratory outpatient setting.

#### Intervention group

Participants in the intervention group received nurse-led shared decision-making support in addition to routine physician care. The intervention followed the three-step shared decision-making model and comprised choice talk, option talk, and decision talk, followed by individualized health guidance [16].

Choice talk was conducted by the consulting respiratory physician as part of routine care. Based on the patient’s medical history and pulmonary nodule imaging characteristics, the physician introduced clinically appropriate management options, provided recommendations for consideration, and answered the patient’s questions. This stage lasted approximately 10 min.

After completion of all required examinations, the patient was referred to a trained nurse, who conducted option talk and decision talk in a separate, quiet consultation room. During option talk, the nurse assessed the patient’s information needs and used the decision aid to explain pulmonary nodules and the available management options. Scheduled CT surveillance, biopsy, and surgical resection were presented in terms of procedures, potential benefits, risks, limitations, and diagnostic performance. The information was tailored to the patient’s clinical situation and level of understanding and was delivered objectively and neutrally.

During decision talk, the nurse guided the patient through the values-clarification component of the decision aid. Patients rated the importance of factors related to the benefits and risks of each option and discussed their concerns, expectations, and preferences. The nurse clarified misunderstandings and supported the patient in identifying a preferred management option. The treating physician was consulted when further clinical clarification was required. Family members were encouraged to participate when present. Patients who remained undecided were allowed additional time and were contacted approximately 1 week later. The nurse-led consultation lasted approximately 20 min.

After the decision-making process, the nurse provided individualized guidance on the selected management plan, follow-up arrangements, lung cancer risk reduction, and healthy lifestyle behaviours. Current smokers also received brief smoking cessation support. For ethical reasons and in line with similar study designs [17], outcome measures were assessed only after completion of the intervention.

#### Control group

Participants in the control group received routine care only. After the physician consultation, nurses provided practical instructions according to the selected management option, including the timing of CT surveillance, preparation for biopsy or surgery, and hospital appointment and admission procedures. Participants in the control group did not receive the structured decision aid, formal values clarification, or nurse-led option talk and decision talk. Current smokers received brief smoking cessation advice in accordance with relevant guidelines.

#### Standardisation and intervention fidelity

All intervention nurses received the same standardized training, followed the same structured programme, and used the same finalized decision aid. Each consultation followed a predefined sequence and included the same core information and values-clarification components. The consultation setting, timing, duration, and physician-to-nurse referral process were standardized after the pilot study.

Nurses were instructed to present all management options in a balanced and non-directive manner and to avoid expressing personal preferences. Clinical uncertainties were referred to the treating physician. These procedures were implemented to minimize variation among nurses and maintain consistency in intervention delivery.

### Outcomes

#### Primary outcome

Decisional conflict among patients with pulmonary nodules was assessed at T1 using the Chinese version of the Decisional Conflict Scale (DCS). The original scale was developed by O’Connor [18] and was subsequently translated and culturally adapted into Chinese by Li Yu [19]，The Chinese version of the DCS consists of 16 items across three dimensions, with higher scores indicating greater decisional conflict. The scale has demonstrated good reliability and validity in the Chinese population, with a Cronbach’s α of 0.897 and a content validity index of 0.950 [20].

#### Secondary outcomes

##### Psychological distress

Psychological distress was assessed using the Impact of Event Scale (IES) at T1, T2, and T3. Assessments at T2 and T3 were limited to participants who did not undergo biopsy or surgical intervention. The IES was originally developed by Horowitz et al. [21], and was subsequently translated and culturally adapted into Chinese by Zhao Chengzhi and colleagues [22]. The scale consists of 15 items across two dimensions and is scored on a 5-point Likert scale ranging from 0 to 4, yielding a total score of 0–75 [23]. Higher scores indicate greater psychological distress. Total scores of ≤ 25 indicate mild or lower levels of distress, whereas scores of ≥ 26 indicate moderate or higher levels of distress. The Chinese version of the IES has demonstrated good reliability, with Cronbach’s α coefficients of 0.90 for the total scale and 0.88 and 0.81 for the two subscales, respectively [22].

##### Satisfaction with participation in decision-making

Satisfaction with participation in medical decision-making was assessed at T1 using the Medical Decision-Making Participation Satisfaction Scale developed by Xu Xiaolin and colleagues [24]. The scale comprises 16 items across four dimensions: information, communication and negotiation, decision-making, and overall satisfaction and confidence. The scale has demonstrated good internal consistency, with a Cronbach’s α coefficient of 0.899. All items are rated on a 5-point Likert scale. Item and total scores were transformed into standardized scores on a 100-point scale based on the original raw scores, with higher scores indicating greater satisfaction with participation in decision-making.

##### Pulmonary nodules knowledge score

Pulmonary nodule-related knowledge was assessed at T1, T2, and T3 using a seven-item questionnaire developed by the research team. The questionnaire was designed to evaluate patients’ basic knowledge of pulmonary nodules and their understanding of the management options addressed in the decision aid and shared decision-making intervention, including the potential benefits, risks, limitations, and diagnostic performance of each option.

Each item was scored as 10 points for a correct response and 0 points for an incorrect or “do not know” response. The total score ranged from 0 to 70, with higher scores indicating greater pulmonary nodule-related knowledge. For psychometric analyses, item responses were coded dichotomously as correct = 1 and incorrect or “do not know” = 0.

Content validity was evaluated by 10 experts. The item-level content validity index ranged from 0.80 to 1.00, and the scale-level content validity index based on the average method was 0.91, indicating acceptable content validity. In the present sample, the Kuder–Richardson Formula 20 coefficient was 0.750. Corrected item-total correlations ranged from 0.355 to 0.594, and item difficulty indices, expressed as the proportions of correct responses, ranged from 0.367 to 0.684. The items and response options of the Pulmonary Nodule Knowledge Questionnaire are presented in Table S3. Detailed content validity, reliability, and item-level analyses are presented in Tables S4 and S5.

##### Treatment adherence

Treatment adherence was evaluated by comparing patients’ initial decisions with their actual management choices. Follow-up interviews and reviews of electronic medical records were conducted to determine whether patients adhered to the management plan corresponding to their decision. Adherence to CT surveillance was defined as completion of follow-up chest CT within the scheduled time frame. To account for delays related to the COVID-19 pandemic or personal factors, a grace period of 30 days beyond the planned date was allowed. Adherence to biopsy was defined as undergoing biopsy within 1 month after the decision, and adherence to surgical treatment was defined as completion of surgery within 1 month after the decision.

### Sample size

The sample size was estimated using both a two-sample comparison of means and a repeated-measures design, and the larger estimate was adopted as the final sample size requirement. For the two-sample comparison of means, the following formula was used: *n* = 2(*U*_*α*_+*U*_*β*_)^2^ σ^2^ /δ^2^, where n represents the required sample size per group, and *U*_*α*_ and *U*_*β*_ represent the critical values corresponding to type I and type II error rates, respectively. With α set at 0.05 and β at 0.10, *U*_*α*_ = 1.96 and *U*_*β*_ = 1.282. The expected between-group difference in the primary outcome, decisional conflict, was δ = 18.28. The standard deviation was represented by σ, and the larger standard deviation of 22.26 from the two groups was used. Based on this calculation, 31 participants were required per group.

Sample size estimation was also performed using a repeated-measures design with the same α and β values. The design included two groups and three repeated measurements, assuming an effect size f of 0.2 and an intra-subject correlation coefficient of 0.4. Calculation using G*Power indicated that 33 participants were required per group. To ensure adequate statistical power, both estimation methods were considered, and the larger estimate was adopted. Accordingly, at least 33 participants per group were required. Considering an anticipated attrition rate of approximately 20%, the sample size was increased to at least 42 participants per group.

### Quality control

To ensure standardization and consistency in intervention delivery, the researchers reviewed domestic and international clinical guidelines and expert consensus statements on pulmonary nodule management before study implementation. They also participated in outpatient clinical practice with respiratory physicians to become familiar with care pathways, common patient concerns, and physician–patient communication strategies for patients with pulmonary nodules. In addition, the researchers completed a publicly available decision support skills training course offered by The Ottawa Hospital and obtained certification, which helped ensure that they had the theoretical knowledge and practical skills required to deliver shared decision-making support.

During data collection, standardized survey instructions were used to minimize subjective influence on participants. Completed questionnaires were reviewed and collected on site by the researchers. If missing items were identified, participants were contacted promptly for clarification to ensure data completeness and accuracy.

### Statistical analysis

Statistical analyses were performed using R software (version 4.1.2). The normality of continuous variables was assessed using the Shapiro–Wilk test. Normally distributed continuous variables were expressed as mean ± standard deviation, and categorical variables were presented as frequencies and percentages. Between-group comparisons were performed using independent-samples t tests for continuous variables and the chi-square test or Fisher’s exact test for categorical variables, as appropriate.

The primary outcome was decisional conflict. An exploratory mediation analysis was conducted to examine the potential mechanisms underlying the effect of nurse-led shared decision-making support on decisional conflict. Guided by the shared decision-making framework and previous decision aid research, pulmonary nodule-related knowledge and satisfaction with participation in decision-making were prespecified as clinically relevant potential mediators. The intervention was hypothesized to reduce decisional conflict by improving patients’ understanding of the available management options and strengthening their involvement in the decision-making process [25, 26]. Mediation was examined using multiple linear regression following the causal-steps approach described by Baron and Kenny [27]. First, the total effect of the intervention on decisional conflict was estimated by regressing decisional conflict on intervention allocation. Second, each proposed mediator was regressed separately on intervention allocation to determine whether the intervention was associated with the mediator. Third, decisional conflict was regressed simultaneously on intervention allocation and the proposed mediators. Findings were considered consistent with mediation when a proposed mediator was significantly associated with decisional conflict and the coefficient for intervention allocation was attenuated or became statistically non-significant after inclusion of the mediator.

Secondary outcomes included psychological distress and pulmonary nodule-related knowledge. Given the correlation inherent in longitudinal data and the potential presence of missing values, generalized estimating equations were used to examine between-group differences in outcome changes over time. The models included group, time, and group × time interaction terms, and all analyses were conducted according to the intention-to-treat principle. Outcome variables were specified as dependent variables, with intervention group, time, and the group × time interaction entered as independent variables. A significant main effect of group indicated overall differences between groups regardless of time. A significant main effect of time indicated changes in outcomes over time, and a significant group × time interaction indicated differential trends in outcome changes between groups across time points. Estimated marginal means and pairwise comparisons were obtained to compare within-group and between-group differences in outcome scores at different time points. Cohen’s d was calculated to quantify effect sizes, with values < 0.5 indicating small effects, 0.5–0.8 indicating moderate effects, and ≥0.8 indicating large effects.

## Results

### Participant enrollment and follow-up

**Fig. 1:**
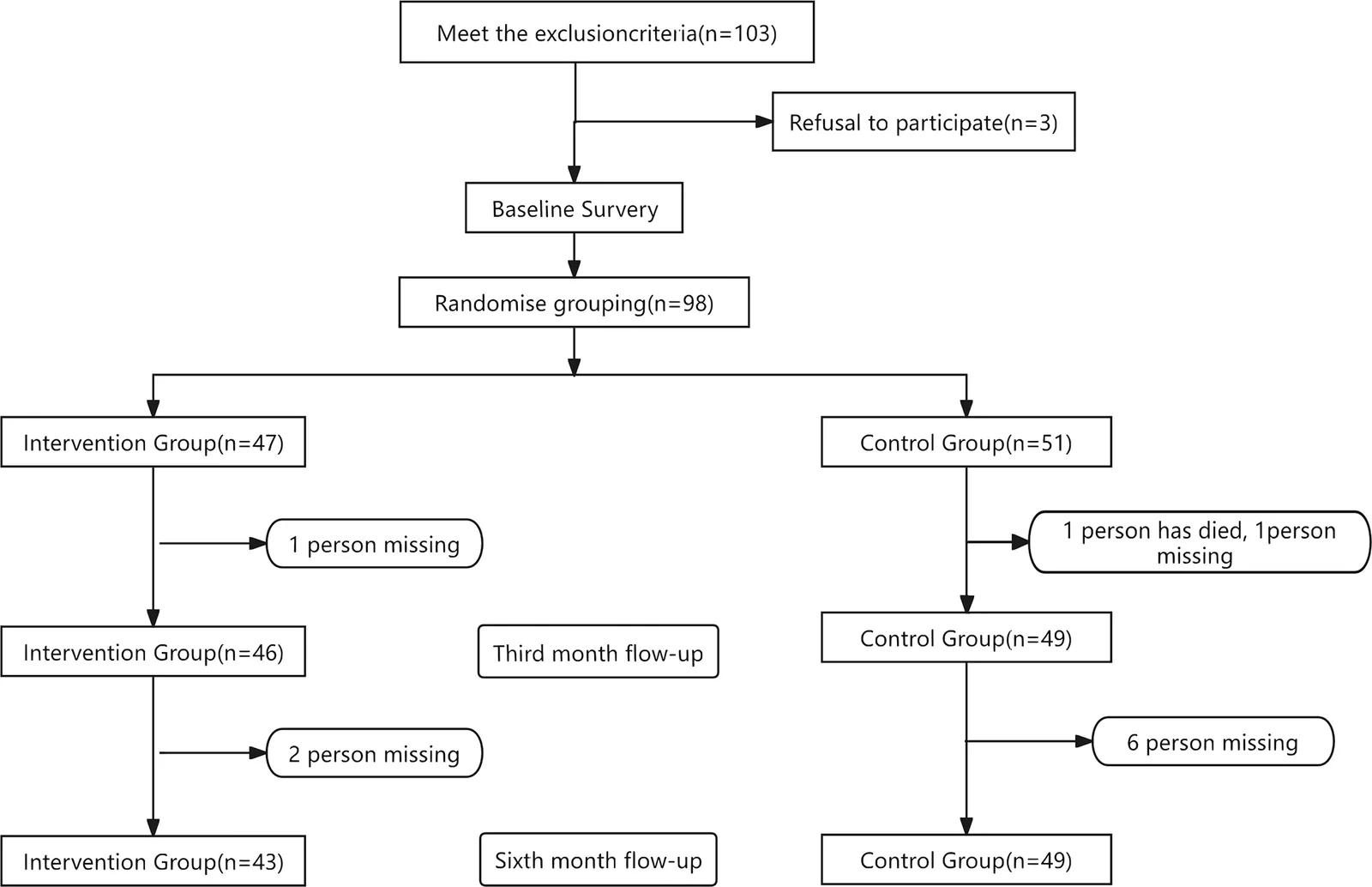
Participant flow diagram

At baseline (T1), 101 individuals met the inclusion criteria. Of these, three declined to participate, and the remaining 98 participants were randomly allocated to the intervention group (*n* = 47) or the control group (*n* = 51). At the 3-month follow-up (T2), one participant in each group was lost to follow-up, and one participant in the control group died. At the 6-month follow-up (T3), three participants in the intervention group and six participants in the control group were lost to follow-up. The participant enrolment and follow-up process is shown in Fig. 1

### Baseline characteristics of participants

A total of 98 participants were included at baseline. Of these, 57 were female (58.2%), and the mean age was 53.08 ± 12.27 years. Most participants reported a per capita monthly household income of more than 4,000 RMB (71.5%), were non-smokers (72.4%), and had no long-term occupational or environmental exposure to dust (90.8%). Approximately half of the participants (52.0%) reported family involvement in decision-making regarding pulmonary nodule management. Most participants (62.2%) wished physicians to better understand their perspectives during the decision-making process, whereas 33.7% believed that medical decisions could be made entirely by physicians. Baseline characteristics, including demographic characteristics, pulmonary nodule-related risk factors, and decision-making preferences, did not differ significantly between the intervention and control groups, indicating good comparability between groups.(Table 1)

**Table 1:** Comparison of general data between the two groups before intervention

Variable	$\bar x \pm s$*/n* (%)		*t/x*^*2*^	P
	Control Group (*n=*51)	Intervention Group (*n* = 47)		
Gender			0	1
Male	21 (41.2)	20 (42.6)		
Female	30 (58.8)	27 (57.4)		
Age	52.84 (12.10)	53.34 (12.58)	−0.199	0.842
Educational level^1^			-	0.587
Undergraduate and above	11 (21.6)	9 (19.1)		
Junior college	6 (11.8)	3 (6.4)		
Junior high school or vocational school	5 (9.8)	10 (21.3)		
Middle school	20 (39.2)	17 (36.2)		
Elementary school and blow	9 (17.6)	8 (17.0)		
Marital status^1^			-	1
Unmarried or other	4 (7.8)	3 (6.4)		
Married	47 (92.2)	44 (93.6)		
Occupational status^1^			-	0.504
Individual or Business or Enterprise or Government service	9 (17.6)	12 (25.5)		
Workers	7 (13.7)	6 (12.8)		
Homemakers	3 (5.9)	5 (10.6)		
Professionals (technology, healthcare, and education)	5 (9.8)	4 (8.5)		
Retired persons	5 (9.8)	0 (0.0)		
Farmers	13 (25.5)	11 (23.4)		
Administrative staff	3 (5.9)	4 (8.5)		
Others	6 (11.8)	5 (10.6)		
Long-term exposure to dust^1^			-	0.407
No	48 (94.1)	41 (87.2)		
Yes	3 (5.9)	6 (12.8)		
Income level (CNY)^1^			1.256	0.74
≤3,000	8 (15.7)	5 (10.6)		
3001–4000	9 (17.6)	6 (12.8)		
4001–5000	15 (29.4)	17 (36.2)		
≥5,001	19 (37.3)	19 (40.4)		
Residence^1^			-	0.88
Prefecture-level city	18 (35.3)	13 (27.7)		
Rural	2 (3.9)	2 (4.2)		
Provincial capital city	18 (35.3)	19 (40.4)		
County-level area	13 (25.5)	13 (27.7)		
Smoking status^1^			-	0.845
Never	38 (74.5)	33 (70.2)		
Current	9 (17.6)	9 (19.2)		
Former	4 (7.9)	5 (10.6)		
Cancer in first-degree relatives^1^			0.204	0.651
No	44 (86.3)	38 (80.9)		
Yes	7 (13.7)	9 (19.1)		
Family involvement in decision-making^1^			0.151	0.698
No	23 (45.1)	24 (51.1)		
Yes	28 (54.9)	23 (48.9)		
Decision-making preference^1^			-	0.181
Doctor-led	16 (31.4)	17 (36.2)		
Patient-led	4 (7.8)	0 (0.0)		
Shared decision-making	31 (60.8)	30 (63.8)		

Note: ^1^ Fisher’s exact test

### Effects of shared decision-making support on decisional conflict and underlying mechanisms

Post-intervention decisional conflict scores were lower in the intervention group than in the control group for both the total score and all subdomains, including information and values clarity, decision support and effectiveness, and decisional uncertainty (Table 2).

**Table 2:** Total and subscale scores of the decisional conflict scale in the two groups

Variable	$\bar x \pm s$		t	P
	Control Group	Intervention Group		
Total decisional conflict score	48.02 ± 19.93	22.87 ± 17.49	6.579	<0.001
Informed and values clarity	20.44 ± 8.13	8.34 ± 7.18	7.818	<0.001
Support and effective decision-making	22.09 ± 11.99	11.67 ± 9.19	4.849	<0.001
Decision uncertainty	5.33 ± 2.93	2.79 ± 2.50	4.619	<0.001

**Fig. 2:**
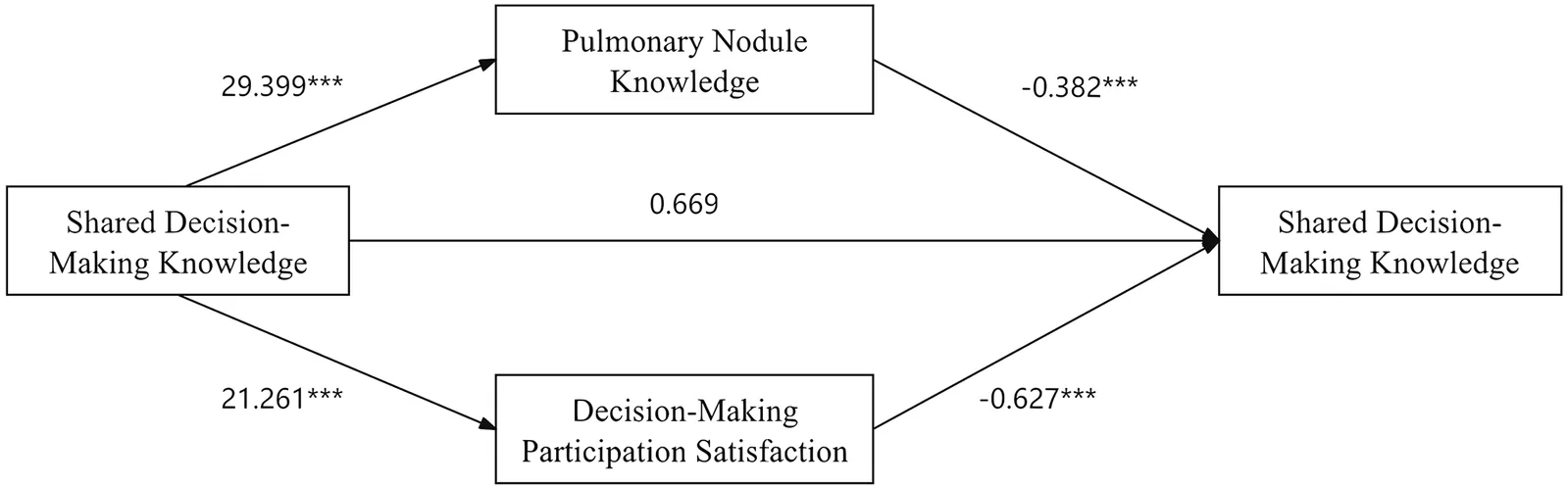
Pathway of shared decision-making support on decision conflict

An exploratory mediation analysis was conducted to examine the mechanisms through which the intervention influenced decisional conflict. Based on the causal-steps approach proposed by Baron and Kenny [27], mediation analyses were performed to assess whether the effect of shared decision-making support on decisional conflict was mediated by pulmonary nodule-related knowledge and satisfaction with participation in decision-making. The intervention had statistically significant effects on decisional conflict (B = −25.05, SE = 3.87, 95% CI −32.74 to −17.36, *p* < 0.001), pulmonary nodule-related knowledge (B = 29.40, SE = 3.26, 95% CI 22.94 to 35.86, *p* < 0.001), and satisfaction with participation in decision-making (B = 21.26, SE = 3.03, 95% CI 15.24 to 27.28, *p* < 0.001) (Table 3). After pulmonary nodule-related knowledge and satisfaction with participation in decision-making were entered into the regression model, the effect of the intervention on decisional conflict was no longer statistically significant (*p* = 0.883). These findings suggest that the effect of shared decision-making support on decisional conflict was mediated through pulmonary nodule-related knowledge and satisfaction with participation in decision-making. The mediation pathways are shown in Fig. 2.

**Table 3:** Stepwise mediation analysis of the mechanisms underlying the effect of the intervention on decisional conflict

	B	95%CI	SE	t	P
**Dependent variable: **Decisional conflict					
Group (intervention)	−25.05	(−32.735, −17.364)	3.872	−6.469	<0.0001
**Dependent variable: **Pulmonary nodule–related knowledge					
Group (intervention)	29.399	(22.935, 35.863)	3.257	9.028	<0.0001
**Dependent variable: **Satisfaction with decision-making involvement					
Group (intervention)	21.261	(15.241, 27.281)	3.033	7.011	<0.0001
**Dependent variable: **Decisional conflict					
Group (intervention)	0.669	(−8.364, 9.702)	4.548	0.147	0.883
Satisfaction with decision-making involvement	−0.627	(−0.835, −0.418)	0.105	−5.971	<0.0001
Pulmonary nodule–related knowledge	−0.382	(−0.576, −0.187)	0.098	−3.9	0.0002
Psychological distress	0.131	(−0.054, 0.316)	0.093	1.408	0.163
Patient decision (biopsy)	0.8	(−6.665, 8.265)	3.758	0.213	0.832
Patient decision (surgery)	−3.067	(−15.089, 8.956)	6.053	−0.507	0.614

### Effects of shared decision-making support on pulmonary nodules knowledge

Generalized estimating equations were used to examine changes in pulmonary nodule-related knowledge scores over time between the two groups. As shown in Table S6, he main effect of group was statistically significant (*χ*^*2*^ = 48.138, *p* < 0.001), indicating an overall difference in pulmonary nodule-related knowledge scores between the intervention and control groups. In addition, Table S7 shows a significant group × time interaction at T2 (B = 8.656, *p* = 0.031), suggesting that changes in pulmonary nodule-related knowledge scores from T1 to T2 differed between groups.

In the control group, pulmonary nodule-related knowledge scores were higher at T2 (mean difference = 6.99, Cohen’s d = 0.40) and T3 (mean difference = 9.09, Cohen’s d = 0.50) than at T1, whereas scores at T3 were comparable to those at T2 (mean difference = 2.10, Cohen’s d = 0.10). In contrast, pulmonary nodule-related knowledge scores in the intervention group showed minimal variation across the three time points, with Cohen’s d values of 0.08, 0.01, and 0.06, respectively. Between-group comparisons showed that knowledge scores were consistently higher in the intervention group than in the control group at T1 (mean difference = 30.09, Cohen’s d = 1.80), T2 (mean difference = 21.44, Cohen’s d = 1.20), and T3 (mean difference = 20.09, Cohen’s d = 1.10), as shown in Table S8 and Fig. S1.

### Effects of shared decision-making support on pulmonary nodule-related psychological distress

Generalized estimating equations were used to evaluate the effect of shared decision-making support on psychological distress. As shown in Table S9, the main effects of group and time and the group × time interaction were statistically significant (*p* < 0.001), indicating between-group differences, changes over time, and differential patterns of change in psychological distress scores between the two groups. As shown in Table S10, significant time effects were observed at T2 (B = −11.63, *p* < 0.001) and T3 (B = −15.86, *p* < 0.001), indicating that psychological distress scores changed significantly at these time points compared with baseline.

Pairwise comparisons of estimated marginal means showed that psychological distress scores in both groups were lower at T2 and T3 than at T1, and scores at T3 were further reduced compared with T2. Psychological distress scores were lower in the intervention group than in the control group at all three time points. Between-group differences were statistically significant at T1 (mean difference = −7.34, Cohen’s d = 0.46) and T2 (mean difference = −11.35, Cohen’s d = 1.18), with the largest difference observed at T2. At T3, although the intervention group continued to show lower psychological distress scores (mean difference = −3.95, Cohen’s d = 0.33), the between-group difference was no longer statistically significant. These results are presented in Table S11 and Fig. S2.

### Effects of shared decision-making support on patients’ decisions and treatment adherence

Patients’ actual post-intervention management choices differed between the two groups. In the intervention group (*n* = 47), 73.3% of patients completed at least one recommended diagnostic procedure, compared with 48.9% in the control group (*n* = 51). Conversely, 26.7% of patients in the intervention group did not undergo any diagnostic tests, compared with 51.1% in the control group (χ^2^ = 4.766, *p* = 0.029; Table S12).

These findings suggest that shared decision-making support may improve adherence to recommended diagnostic procedures. The absolute between-group difference in adherence was 24.4% points, indicating a clinically meaningful difference. This suggests that shared decision-making support may not only improve patients’ understanding and engagement but also facilitate adherence to planned pulmonary nodule management.

## Discussion

Using a randomized controlled design, this study evaluated the effectiveness of nurse-led shared decision-making support delivered with a decision aid in pulmonary nodule management. To our knowledge, this is among the first studies to examine the effects of nurse-led shared decision-making support on decision quality and related outcomes in patients with pulmonary nodules [12, 14, 28]. The findings showed that the intervention reduced decisional conflict and improved pulmonary nodule-related knowledge, satisfaction with participation in decision-making, psychological distress, and treatment adherence. Exploratory mediation analysis further suggested that the reduction in decisional conflict was mainly explained by improvements in patients’ knowledge and satisfaction with participation in decision-making.

### Effect of shared decision-making support on patients’ decisional conflict

This study found that nurse-led shared decision-making support based on a decision aid significantly reduced decisional conflict among patients with pulmonary nodules. This finding is consistent with previous evidence showing that shared decision-making interventions can improve decision quality in preference-sensitive clinical contexts [17]. By providing structured information and decision support, shared decision-making may reduce uncertainty when patients are faced with multiple management options and increase their confidence in decision-making.

The effect of the intervention on decisional conflict may be explained by improvements in pulmonary nodule-related knowledge and satisfaction with participation in decision-making. After the initial detection of pulmonary nodules, it is often difficult to determine benignity or malignancy immediately and accurately [29]. Patients therefore need to choose among CT surveillance, biopsy, and surgical resection, each of which involves different benefits, risks, and uncertainties [30]. Limited understanding of pulmonary nodule-related information may further increase decisional conflict [9]. In routine practice, however, physicians may have limited time to provide detailed and individualized information [31]. In this context, nurse-led decision support may complement physician consultations by providing structured explanations, clarifying patients’ concerns, and supporting informed decision-making [32].

Decision aids are important tools for implementing shared decision-making because they present disease- and management-related information in a standardized and understandable format and help patients clarify their risk–benefit preferences [33, 34]. In the present study, the decision aid and nurse-led consultation may have helped patients understand available management options and participate more actively in the decision-making process. This may explain the observed improvement in satisfaction with participation in decision-making. Previous studies have also shown that information support and emotional support from healthcare professionals can improve patient engagement, strengthen physician–patient relationships, and reduce decisional conflict [35–38].

### Effect of shared decision-making support on patients’ knowledge of pulmonary nodules

Insufficient knowledge of pulmonary nodules and their management options is an important barrier to informed decision-making. Patients may misinterpret pulmonary nodules as lung cancer, leading to unnecessary fear and anxiety. One important goal of shared decision-making support is to help patients understand key information and make informed decisions [39]. In this study, nurse-led shared decision-making support systematically guided patients to understand the benefits, risks, limitations, and diagnostic value of different management options. The intervention significantly improved pulmonary nodule-related knowledge, which is consistent with previous findings showing that structured educational materials can improve patients’understanding of pulmonary nodules [40].

The longitudinal pattern of knowledge scores also warrants attention. Knowledge scores in the intervention group remained relatively stable after T1, possibly because knowledge was first assessed immediately after the structured nurse-led consultation, when participants had already achieved relatively high scores. This may reflect a ceiling effect and also suggests that the knowledge gained from the intervention was largely maintained during follow-up. By contrast, the delayed increase in the control group may be partly explained by natural information acquisition after the initial consultation, including repeated clinical contact, CT follow-up, communication with family members, and independent information seeking from the internet or other healthcare resources [41]. Nevertheless, knowledge scores in the control group remained lower than those in the intervention group at all time points, supporting the earlier and sustained advantage of nurse-led shared decision-making support in improving pulmonary nodule-related knowledge.

### Effect of shared decision-making support on patients’ psychological distress

Psychological distress is common among patients with incidentally detected pulmonary nodules and may persist during follow-up [31]. Some patients may misinterpret pulmonary nodules as lung cancer, resulting in anxiety and fear. In this study, patients in the intervention group had lower psychological distress scores than those in the control group, and their distress declined more rapidly during follow-up. These findings suggest that shared decision-making support may help alleviate psychological distress in patients with pulmonary nodules.

Several mechanisms may explain this effect. First, the decision aid provided clear and structured information, which may have reduced misunderstandings about medical terminology, malignancy risk, and management options. Second, nurse-led communication created an opportunity for patients to express concerns and clarify uncertainties. Third, shared decision-making encouraged communication between patients and family members. In the Chinese cultural context, family members often provide important emotional and decision-related support during unexpected health events [42]. Through joint discussion of the benefits and risks of different options, patients may obtain both informational and emotional support, thereby reducing psychological distress.

Psychological distress decreased over time in both groups, which is consistent with previous findings [43]. This may be related to patients’ gradual adaptation during follow-up and their active information seeking about pulmonary nodules through clinical contact, books, the internet, family members, or other healthcare resources [41]. The increase in pulmonary nodule-related knowledge scores in the control group at T2 also supports this possible explanation.

### Effect of shared decision-making support on patients’ treatment adherence for pulmonary nodules

This study found that approximately half of the patients in the control group did not complete any follow-up examinations within 6 months, indicating suboptimal adherence to pulmonary nodule management. Previous studies have also reported non-adherence during pulmonary nodule follow-up [31, 43, 44]. In contrast, patients in the intervention group showed better adherence to recommended diagnostic procedures, suggesting that nurse-led shared decision-making support may facilitate adherence to planned management.

This effect may be related to improved knowledge, reduced uncertainty, and greater satisfaction with decision participation. Psychological distress has been reported to negatively affect adherence to screening and follow-up behaviours [45]. By addressing patients’ concerns, clarifying management options, and supporting preference-sensitive decisions, shared decision-making may reduce distress and strengthen trust in healthcare providers. These factors may help patients translate their decisions into actual management behaviours. This finding is consistent with evidence that shared decision-making can improve adherence to lung cancer screening [46].

### Implications for practice and future research

As lung cancer screening becomes more widespread, increasing numbers of individuals will be identified with pulmonary nodules. This highlights the need for structured support models for patients with pulmonary nodules. Nurses can play an important role in this process by providing ongoing informational and emotional support in addition to routine physician recommendations. Using decision aids, nurses can help patients understand pulmonary nodules, compare management options, clarify personal preferences, and participate more actively in decision-making.

The findings of this study suggest that nurse-led shared decision-making support may improve pulmonary nodule-related knowledge, satisfaction with participation in decision-making, psychological distress, and adherence to planned management. However, some patients still showed non-adherence, indicating the need for more continuous and comprehensive follow-up strategies. Future studies should explore how shared decision-making can be integrated into routine pulmonary nodule care and how nurses’ competencies in decision support can be strengthened. The role of the “shared decision-making nurse” has been proposed in oncology and may provide a useful model for supporting patients with pulmonary nodule [47]. Future research should also develop and evaluate competency frameworks to improve nurses’ decision-support capabilities and promote high-quality, patient-centred care [48, 49].

### Limitations

This study has several limitations. First, this was a single-centre randomized controlled trial conducted in the respiratory outpatient department of a tertiary hospital in China, with a relatively small sample size. Therefore, the generalisability and transferability of the findings should be interpreted with caution. Cultural and healthcare system factors may influence the implementation and effectiveness of nurse-led shared decision-making support. In the Chinese context, family members often play an important role in medical decision-making, patients may be more familiar with physician-led recommendations, and nurses’ independent role in decision support is still developing. In addition, differences in outpatient workflow, consultation time, nurse staffing, nursing scope of practice, pulmonary nodule management pathways, and access to follow-up imaging or biopsy services may affect the feasibility of applying this intervention in other settings. Future multicentre studies involving diverse healthcare institutions, cultural contexts, and patient populations are needed.

Second, the follow-up duration was relatively short. Although psychological distress, pulmonary nodule-related knowledge, and treatment adherence were assessed during follow-up, the long-term effects of shared decision-making support on subsequent management decisions, final treatment outcomes, long-term adherence, and health outcomes remain unclear. Future studies with longer follow-up periods are warranted.

Third, the pulmonary nodule-related knowledge questionnaire was developed by the research team and consisted of only seven dichotomously scored items. Although it was developed based on clinical practice, relevant guidelines, and the content of the decision aid, and demonstrated acceptable content validity and internal consistency in this sample, its psychometric evaluation remains limited. Test–retest reliability, construct validity, criterion validity, and responsiveness to change were not fully assessed. The small number of items and binary response format may have limited the sensitivity of the questionnaire to detect subtle changes in knowledge over time. Because pulmonary nodule-related knowledge was included as a key mediator in the exploratory mediation analysis, the limited psychometric evaluation and potential sensitivity constraints of this measure should be considered when interpreting the mediation findings. Therefore, the mediation results related to knowledge should be interpreted with caution and confirmed in future studies using a more comprehensively validated measure.

## Conclusion

In addition to routine physician care, nurse-led shared decision-making support delivered using a decision aid reduced decisional conflict among patients with pulmonary nodules by improving pulmonary nodule-related knowledge and satisfaction with participation in decision-making. The intervention also showed beneficial effects in reducing psychological distress and improving adherence to planned pulmonary nodule management.

## Electronic supplementary material

Below is the link to the electronic supplementary material.

Supplementary Material 1

## Data Availability

The datasets generated during or analyzed during the current study are available from the corresponding author on reasonable request.
