# Supplementary material for: Effects of nurse-led shared decision-making support based on a decision aid on decisional conflict in patients with pulmonary nodules: a randomized controlled trial
Source: BMC Nurs. 2026 Jul 29;25:1017. doi: 10.1186/s12912-026-05040-w (PMC13700445; doi:10.1186/s12912-026-05040-w)
Supplement: Supplementary file 1 [file 12912_2026_5040_MOESM1_ESM.pdf]

Supplementary Material

Supplementary file list

Item	Description
Table S1	Pilot study outcomes by study group
Table S2	Shared decision-making support programme for pulmonary nodule diagnosis and treatment
Table S3	Pulmonary Nodule Knowledge Questionnaire
Table S4	Content validity of the pulmonary nodule knowledge questionnaire
Table S5	Reliability and item analysis of the pulmonary nodule knowledge questionnaire
Table S6	Generalized estimating equation analysis of pulmonary nodule knowledge scores
Table S7	Parameter estimates for pulmonary nodule knowledge scores
Table S8	Pairwise comparisons of pulmonary nodule-related knowledge scores
Table S9	Generalized estimating equation analysis of psychological distress
Table S10	Parameter estimates for psychological distress
Table S11	Pairwise comparisons of psychological distress scores
Table S12	Patient decision-making and adherence between groups
Figure S1	Trend of pulmonary nodule knowledge scores over time
Figure S2	Trend of psychological distress scores over time
Additional file 1	Pulmonary nodule management decision aid

Formatting note: Statistical symbols, table layout, spacing, and caption format were standardized. Numerical results and substantive content were retained.

Table S1. Pilot study outcomes by study group

Outcome	Control group (n = 10), mean ± SD	Intervention group (n = 10), mean ± SD	*t*	*P*
Decisional conflict	45.16 ± 16.16	26.88 ± 22.26	2.04	0.056
Psychological distress	34.30 ± 14.48	27.00 ± 15.58	1.08	0.292
Satisfaction with participation in decision-making	66.75 ± 18.35	79.31 ± 5.63	−2.07	0.053
Pulmonary nodule-related knowledge	25.01 ± 20.14	41.01 ± 18.53	−1.85	0.081

Note. The pilot sample included 20 patients, with 10 participants in each group. The pilot study was conducted to assess feasibility and refine study procedures rather than to provide a definitive test of intervention effectiveness.

Table S2. Shared decision-making support programme for pulmonary nodule diagnosis and treatment

Stage	Objective	Content	Implementation notes
1. Choice talk (attending physician; approximately 10 min)	Clarify the available options	The physician assesses the patient's medical history and pulmonary nodule imaging characteristics and provides clinically appropriate diagnostic and management recommendations.	This stage follows routine clinical practice. The physician answers questions, while the intervention nurse remains in the consultation room to provide assistance and establish initial rapport.
2. Option talk (nurse; approximately 15 min)	Explain the purpose of the discussion and establish rapport	After the physician consultation, the nurse explains that the discussion aims to provide decision support, improve understanding of pulmonary nodules and available management options, and facilitate a decision consistent with the patient's clinical situation and preferences.	If other hospital examinations are required, the discussion is conducted after these are completed. The consultation takes place in a separate, quiet room to protect privacy. Family participation is encouraged. The nurse clarifies the options under consideration and elicits remaining questions and decision-support needs.
	Improve understanding of pulmonary nodules and reduce anxiety	Using the decision aid and adapting the explanation to the patient's level of understanding, the nurse explains the definition, common causes, epidemiological features, and relationship between pulmonary nodules and lung cancer.	The nurse explains the likelihood of malignancy to reduce unnecessary anxiety while avoiding false reassurance. Misconceptions that pulmonary nodules require no attention or management are corrected appropriately.
	Explain the benefits and risks of each management option	Using the decision aid, the nurse explains the meaning, procedures, purpose, benefits, risks or limitations, and diagnostic performance of relevant options, including CT surveillance, biopsy, and surgical resection.	All options are presented objectively and neutrally. Medical terminology is explained in plain language. Information is tailored to the patient's nodule characteristics and physician recommendations to avoid information overload. Questions are used to identify knowledge gaps and decisional conflict.
	Strengthen willingness to participate in decision-making	The nurse explains that there is no universally correct option and that an appropriate decision should integrate clinical considerations with the patient's values and concerns. The patient is asked about the role they wish to take in decision-making and is encouraged to participate actively.	If a decision has already been made, the nurse explores the reasons and corrects inaccurate understanding. Shared decision-making is explained as a collaborative process involving healthcare professionals, patients, and family members.
3. Decision talk (nurse; approximately 5 min)	Clarify decision preferences	The nurse uses the 'weighing benefits and risks' module of the decision aid to help the patient identify what matters most and clarify attitudes toward each option.	The nurse summarizes the preceding discussion, asks what benefits or risks are most important, and uses a 0–5 rating scale to assess the importance of each factor. The nurse does not express a personal preference.
	Encourage questions and expression of concerns	After preferences have been clarified, the nurse encourages questions, responds promptly, and consults the attending physician when necessary. Misunderstandings are corrected and emotional concerns are acknowledged.	Patient preferences are balanced with the clinical situation. The nurse may restate the patient's concerns and provide additional information when needed.
	Reach a shared decision	After reviewing the options and weighing their benefits and risks, the nurse supports the patient in reaching a decision. If the patient remains undecided, additional time is allowed for discussion with family members, and follow-up contact is made approximately one week later.	The nurse communicates with the physician when clinical clarification is required. Responses remain objective and non-directive. Family members may participate in discussion and health education. Patients receive the decision aid and may access the more detailed electronic version via QR code.
4. Health guidance (nurse; approximately 5 min)	Promote smoking cessation	Current smokers and family members who smoke receive a brief smoking cessation intervention covering smoking-related lung cancer risk, harms of smoking, benefits of cessation, and available cessation services.	Guidance is individualized according to age, sex, smoking history, and lifestyle. During follow-up, the nurse asks about willingness to quit, cessation status, withdrawal symptoms, and medication use.
	Promote healthy lifestyles and appropriate screening	Health education covers reduction of cooking-fume exposure, occupational protection, appropriate physical activity, avoidance of environmental pollution, healthy diet, and evidence-based lung cancer screening.	Screening advice is provided according to age, smoking history, and other risk factors, and eligible patients and family members are encouraged to participate in recommended screening.

Note. The programme was delivered using the same finalized decision aid and predefined sequence. The full decision aid is provided in Additional file 1.

Table S3. Pulmonary Nodule Knowledge Questionnaire

Item	Correct	Incorrect	Don't know
1. Pulmonary nodules are equivalent to lung cance	Correct	Incorrect	Don't know
2. Regular CT follow-up can help determine the nature of pulmonary nodules by monitoring their changes	Correct	Incorrect	Don't know
3. Biopsy can accurately determine whether a nodule is cancerous	Correct	Incorrect	Don't know
4. Biopsy carries a risk of complications	Correct	Incorrect	Don't know
5. Compared with open thoracotomy, thoracoscopic minimally invasive resection of pulmonary nodules allows for faster recovery	Correct	Incorrect	Don't know
6. Surgical resection may also be necessary for benign nodules	Correct	Incorrect	Don't know
7. Malignant nodules can have a favorable prognosis if detected and resected early	Correct	Incorrect	Don't know

Table S4. Content validity of the pulmonary nodule knowledge questionnaire

The content validity of the seven-item pulmonary nodule knowledge questionnaire was evaluated by 10 experts. Ratings of 3 or 4 were coded as 1 (relevant), whereas ratings of 1 or 2 were coded as 0 (not relevant).

Item	N	Minimum	Maximum	I-CVI	SD
CVI1	10	1.00	1.00	1.00	0.000
CVI2	10	1.00	1.00	1.00	0.000
CVI3	10	0.00	1.00	0.90	0.316
CVI4	10	0.00	1.00	0.90	0.316
CVI5	10	0.00	1.00	0.80	0.422
CVI6	10	0.00	1.00	0.80	0.422
CVI7	10	1.00	1.00	1.00	0.000
I-CVI range				0.80–1.00	
S-CVI/Ave				0.91	
S-CVI/UA				0.43	

Note. I-CVI, item-level content validity index; S-CVI/Ave, scale-level content validity index using the average method; S-CVI/UA, scale-level content validity index using universal agreement; SD, standard deviation. S-CVI/Ave was calculated as the mean of the seven I-CVI values, and S-CVI/UA as the proportion of items rated as relevant by all experts.

Table S5. Reliability and item analysis of the pulmonary nodule knowledge questionnaire

Each item was scored as 10 points for a correct response and 0 points for an incorrect or "do not know" response, yielding a total score of 0–70. For psychometric analyses, item responses were coded dichotomously (correct = 1; incorrect or "do not know" = 0).

A. Overall reliability

Valid cases, n (%)	Number of items	KR-20 / Cronbach's alpha	Standardized alpha
98 (100.0)	7	0.750	0.749

B. Item-level statistics

Item	Difficulty index (proportion correct)	SD	Corrected item-total correlation	Cronbach's alpha if item deleted
K1	0.684	0.467	0.379	0.738
K2	0.602	0.492	0.588	0.693
K3	0.510	0.502	0.594	0.691
K4	0.571	0.497	0.415	0.731
K5	0.429	0.497	0.355	0.745
K6	0.367	0.485	0.405	0.733
K7	0.541	0.501	0.532	0.705

C. Scale score

Scoring range	Mean	Variance	SD
0–70	37.04	474.70	21.79

Note. KR-20, Kuder–Richardson Formula 20; SD, standard deviation. For dichotomously scored items, Cronbach's alpha is equivalent to KR-20. Corrected item-total correlations ranged from 0.355 to 0.594, and Cronbach's alpha if an item was deleted ranged from 0.691 to 0.745; none exceeded the overall coefficient.

Table S6. Generalized estimating equation analysis of changes in pulmonary nodule knowledge scores over time between two groups

Effect	Wald χ^2	df	*P*
Group	48.138	1	<0.001
Time	2.455	2	0.293
Group × time	4.664	2	0.097

Table S7. Parameter estimates of the generalized estimating equation analysis of pulmonary nodule knowledge scores

Parameter	*B*	SE	95% CI (lower)	95% CI (upper)	Wald χ^2	*P*
Group	-30.092	3.1957	-36.355	-23.828	88.668	<0.001
Time (T2)	-1.667	2.2024	-5.983	2.65	0.573	0.449
Time (T3)	-0.912	2.9083	-6.613	4.778	0.098	0.754
Group × time (T2)	8.656	4.0235	0.77	16.542	4.628	0.031
Group × time (T3)	10.003	5.2192	-0.227	20.232	3.673	0.055

Table S8. Pairwise comparisons of estimated marginal means of pulmonary nodule-related knowledge scores

Item	Mean difference (I–J)	Standard error	*P*	Cohen's d
**Control group**				
T2–T1	6.99	3.367	0.038	0.40
T3–T1	9.09	4.334	0.036	0.50
T3–T2	2.10	2.398	0.381	0.10
**Intervention group**				
T2–T1	−1.67	2.202	0.449	0.08
T3–T1	−0.91	2.908	0.754	0.01
T3–T2	0.75	1.504	0.616	0.06
**Between-group comparison**				
T1 (G1–G2)	30.09	3.196	<0.001	1.80
T2 (G1–G2)	21.44	4.195	<0.001	1.20
T3 (G1–G2)	20.09	4.985	<0.001	1.10

Note. T1 = immediately after the intervention; T2 = 3 months after the intervention; T3 = 6 months after the intervention; G1 = intervention group; G2 = control group.

Table S9. Generalized estimating equation analysis of psychological distress over time between the intervention and control groups

Effect	Wald χ^2	df	*P*
Group	13.818	1	<0.001
Time	127.01	2	<0.001
Group × time	21.092	2	<0.001

Table S10. Parameter estimates from the generalized estimating equation model for psychological distress

Parameter	*B*	SE	95% CI (lower)	95% CI (upper)	Wald χ^2	*P*
Group	7.338	3.4054	0.663	14.012	4.643	0.031
Time (T2)	-11.625	2.0264	-15.597	-7.653	32.91	<0.001
Time (T3)	-15.856	2.6782	-21.105	-10.606	35.049	<0.001
Group × time (T2)	4.009	3.3579	-2.573	10.59	1.425	0.233
Group × time (T3)	-3.383	3.7096	-10.654	3.888	0.832	0.362

Table S11. Pairwise comparisons of estimated marginal means of psychological distress scores

Comparison	Mean difference (I–J)	Standard error	*P*	Cohen's d
**Control group**				
T2–T1	-7.62	2.678	0.004	0.56
T3–T1	-19.24	2.567	<0.001	1.35
T3–T2	-11.62	1.393	<0.001	1.06
**Intervention group**				
T2–T1	-11.62	2.026	<0.001	0.79
T3–T1	-15.86	2.678	<0.001	1.06
T3–T2	-4.23	0.897	<0.001	0.58
**Between-group comparison**				
T1 (G1–G2)	-7.34	3.405	0.031	0.46
T2 (G1–G2)	-11.35	2.212	<0.001	1.18
T3 (G1–G2)	-3.95	2.266	0.081	0.44

Note. T1 = immediately after the intervention; T2 = 3 months after the intervention; T3 = 6 months after the intervention; G1 = intervention group; G2 = control group.

Table S12. Comparison of patient decision-making and adherence between the two groups

Item	Control group (n = 51), n (%)	Intervention group (n = 47), n (%)	χ^2	*P*
**Initial decision**[1]			**-**	**0.225**
CT follow-up	40 (78.4)	30 (63.8)		
Biopsy	9 (17.6)	12 (25.5)		
Surgery	2 (3.9)	5 (10.6)		
**Actual choice**[1]			**-**	**0.152**
CT follow-up	11 (21.6)	18 (38.3)		
Biopsy	8 (15.7)	9 (19. 1)		
Surgery	4 (7.8)	6 (12.8)		
Lost to follow-up[a]	4 (7.8)	2 (12.8)		
No examination performed	24 (47. 1)	12 (25.5)		
**Adherence (n = 92)**			**4.766**	**0.029**
No examination performed	24 (51. 1)	12 (26.7)		
At least one diagnostic option completed	23 (48.9)	33 (73.3)		

Note. [1] Fisher's exact test. [a] The number of patients lost to follow-up in this table differs from that reported in the follow-up section because some patients had already completed relevant examinations before being lost to follow-up.

Fig. S1. Trend of pulmonary nodule knowledge scores at different time points in the intervention and control groups

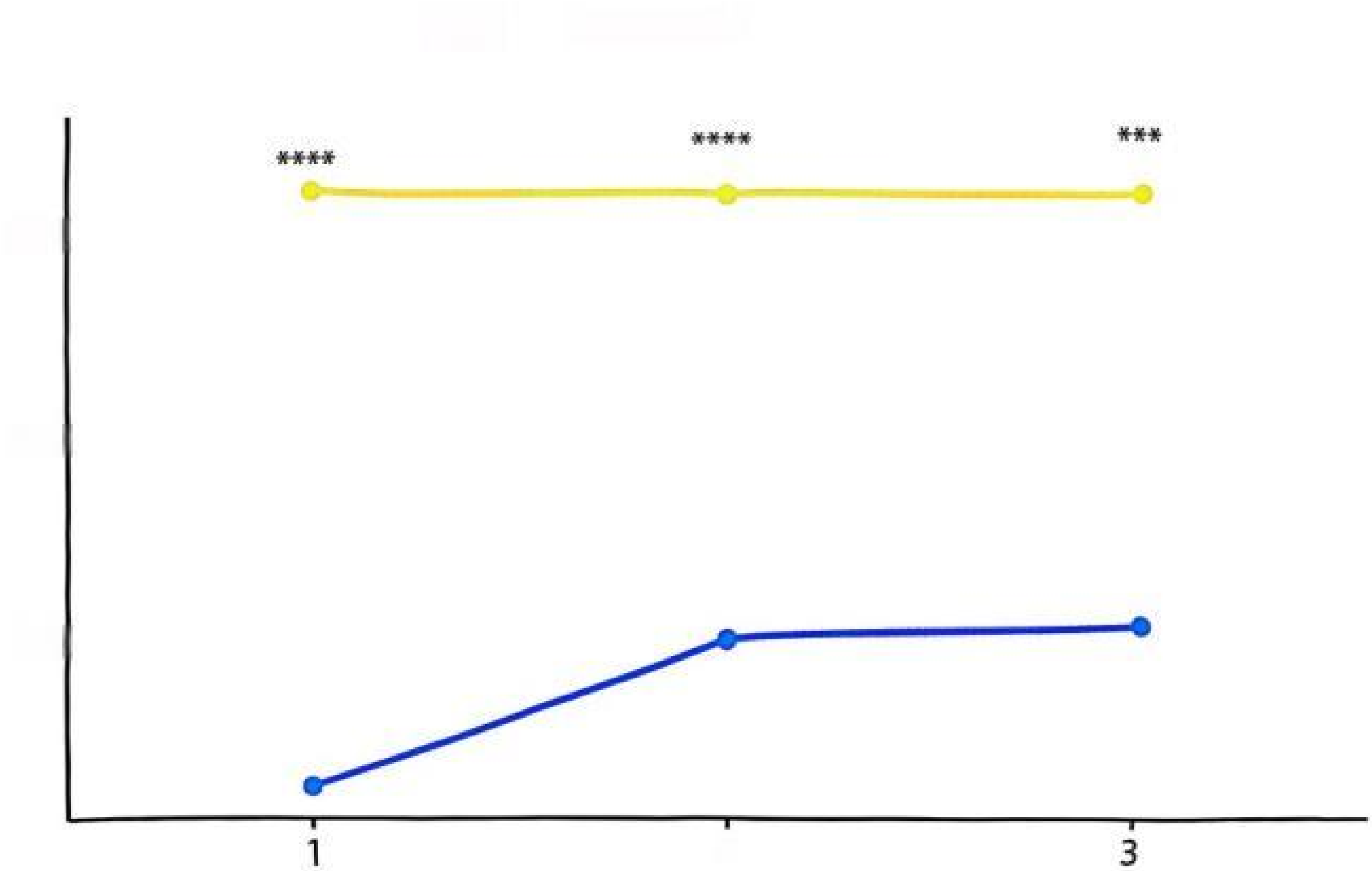

Note. T1 = immediately after the intervention; T2 = 3 months after the intervention; T3 = 6 months after the intervention.

Fig. S2. Trend of psychological distress scores at different time points in the intervention and control groups

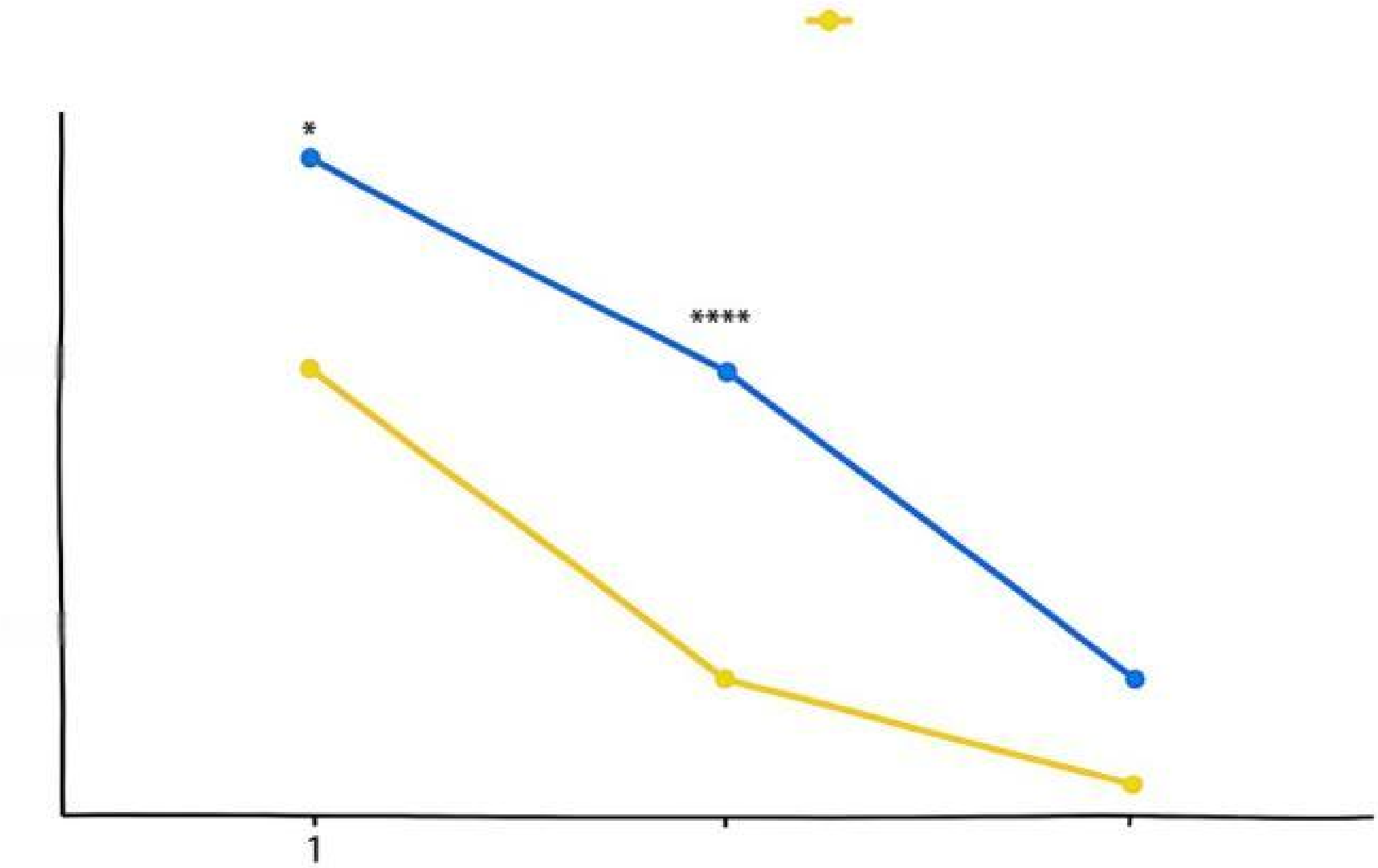

Note. * P<0.05; **** P<0.0001; ns indicates no statistically significant difference.

Additional file 1. Pulmonary nodule management decision aid

UNDERSTANDING PULMONARY NODULES

1. What is a pulmonary nodule?

A pulmonary nodule is'a.round or irregularsolid or subsolidopacity seen on-CT. Itis less than 3 cm in diameter and may occur asa single lesion or as multiple nodules.

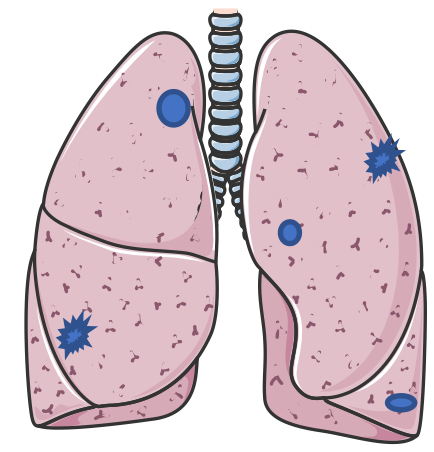

2. What causes pulmonary nodules?

Pulmonary nodules have many possible causes. Common causes include carbon deposition related to smoking or air pollution; tuberculosis or other inflammation; residual changes after chest trauma or pulmonary congestion; benign lung tumours; normal lung tissue; mucus plugs; congenital abnormalities; and pulmonary arteriovenous malformations. A nodule may also represent early-stage lung cancer.

3. How common are pulmonary nodules, and how likely are they to be lung cancer?

Pulmonary nodules are common. They are detected in approximately 26% of people undergoing chest CT during health examinations. Only about 4% are ultimately diagnosed as lung cancer; most are benign.

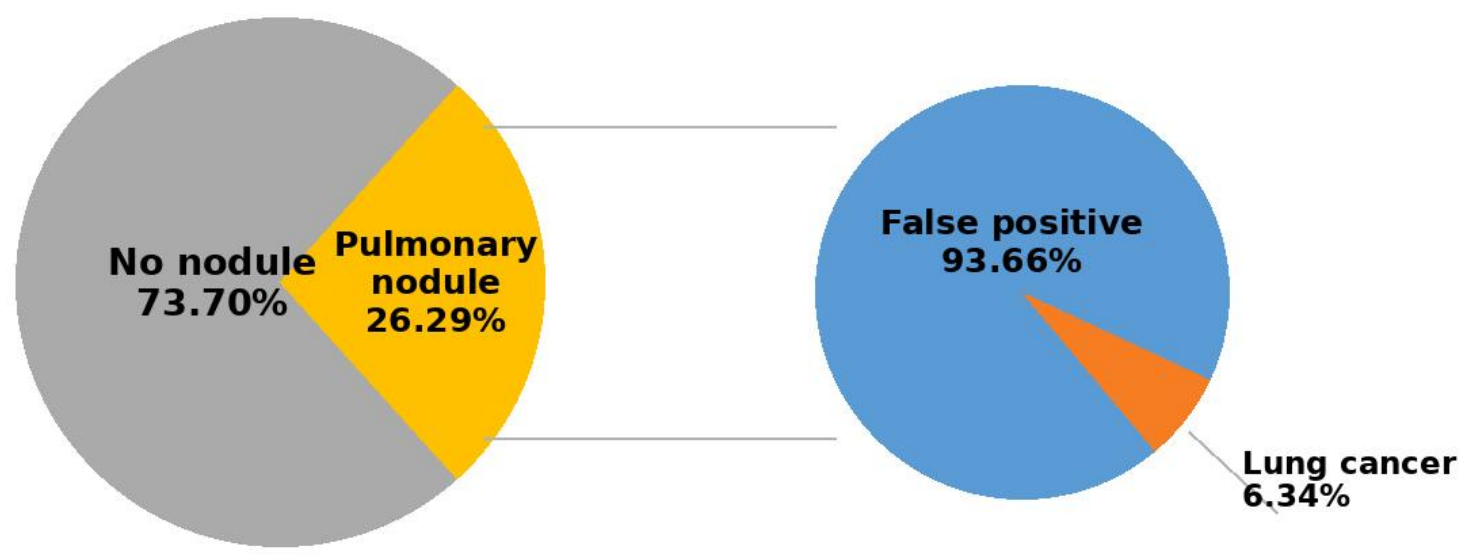

Pulmonary nodules detected in routine chest CT screening

4. What if my pulmonary nodule is lung cancer?

Even when a pulmonary nodule is ultimately diagnosed as lung cancer, it is likely to be at an early stage and generally has a much better prognosis than advanced disease.

5. What should I do now? (Three steps)

Step 1: Understand the available options and their benefits and risks.

Step 2: Weigh the benefits and risks and identify what matters most to you.

Step 3: Make a decision.

Our healthcare team will support you through every step of pulmonary nodule evaluation and management.

1. What options do I have, and what are their benefits and risks?

Category	Option	Description	Advantages	Risks /limitations	Diagnostic performance
CT surveillance	**Scheduled follow-up CT**	Compare serial CT scans to assess nodulechanges and estimate the likelihood ofmalignancy. 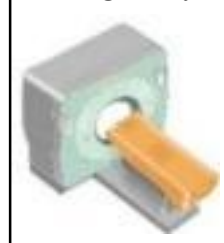	· Non-invasive<br>· Relatively low cost<br>· May help identify other chest conditions	· Repeated radiation exposure<br>· Diagnostic delay while waiting several months (depending on the clinical situation)<br>· Imaging cannot provide a definitive pathological diagnosis	Nodule size measurements may varyby several millimetres.
**Biopsy**	**CT-guided percutaneous lung biopsy**	Under B-mode ultrasound or CT guidance, aneedle is passed through the chest wall toobtain a tissue sample. 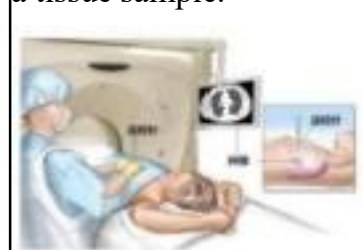	· Relatively low cost<br>· High diagnostic accuracy<br>· May reduce unnecessary removal of benign nodules	· Risk of complications, such as pneumothorax or bleeding<br>· Pathology may still be inconclusive	Sensitivity: approximately 90%
	**Electronic bronchoscopy**	A flexible bronchoscope is advanced throughthe airway to visualize the lesion and obtainsamples. 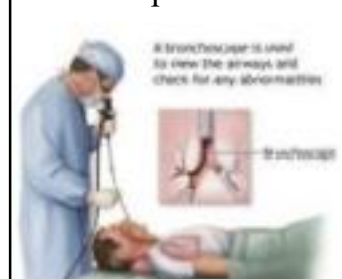	· Low complication rate<br>· May identify other airway disorders<br>· May reduce unnecessary removal of benign nodules	· Limited ability to diagnose peripheral nodules<br>· Risk of complications<br>· Pathology may still be inconclusive	Sensitivity:<br>≤2 cm: 34%<br>>2 cm: 63%
	**Advanced imaae-auided bronchoscopy**	Ultrasound, virtual navigation, orelectromagnetic navigation is used to localizethe lesion and obtain samples. 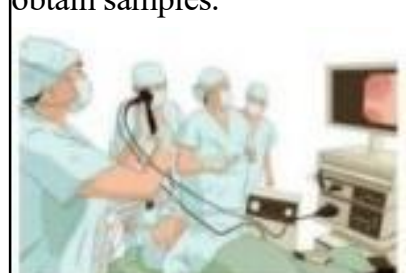	· More accurate localization than conventional bronchoscopy<br>· Can be combined with EBUS/TBLB/TBNA<br>·Low complication rate	· Relatively high cost<br>·A negative result cannot excludemalignancy<br>· Pathology may still be inconclusivee	EBUS: overall sensitivity 72.4%<br>Virtual navigation: diagnostic yield74.17%<br>Electromagnetic navigation: sensitivity77%
**Surgery**	**Thoracoscopic surgical resection**	common procedures include wedge resection, segmentectomy, and lobectomy 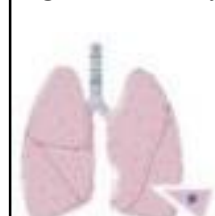	· High diagnostic accuracy<br>· Resection may be curative<br>· If uncomplicated, hospital stay isusually 3-5 days	· Risk of complications<br>· Temporary reduction in lung function<br>· Benign nodules may be unnecessarily removed (11%-34%)	Accuracy may approach 100%. Small or deep nodules may require preoperative localization or intraoperative identification.

2. WEIGHING BENEFITS AND RISKS

	**Please rate how important each factor is to you.**	**Not important Moderately important Very important**
**Follow-up CT in ( ) months**	Make the next decision based on the follow-up CT results.	1 2 3 4 5
	Avoid invasive tests unless they are necessary.	1 2 3 4 5
	I am concerned about complications from biopsy or surgery.	1 2 3 4 5
	**Please rate how important each factor is to you.**	**Not important Moderately important Very important**
**BIOPSY**	I am concerned that waiting for follow-up CT may delay treatment.	1 2 3 4 5
	I do not want surgery before a diagnosis is confirmed.	1 2 3 4 5
	**Please rate how important each factor is to you.**	**Not important Moderately important Very important**
**SURGERY**	I prefer surgical removal regardless of the type of nodule.	1 2 3 4 5
	I do not want to undergo CT examinations too frequently.	1 2 3 4 5

3. MAKING A DECISION

**Your choice:**	
( ) Scheduled follow-up CT	( ) Biopsy
( ) Surgery	( ) Not yet decided

HEALTH GUIDANCE

1. Is my pulmonary nodule related to smoking?

Smokers have 2.77 times the risk of lung cancer compared with non-smokers. The risk increases with the amount and duration of smoking. Exposure to second-hand and third-hand smoke also increases risk.

2. Is it too late to stop smoking?

- It is never too late to stop smoking, and the benefits begin immediately.
- Research suggests that even quitting later in life may reduce the risk of death by 35%.
- Our hospital provides a specialist smoking-cessation clinic, where physicians and nurses offer individualized counselling and guidance. You may also call the smoking-cessation hotline at 400-888-5531.

3. In addition to following my management plan, what else should I do?

Reduce cooking-fume exposure

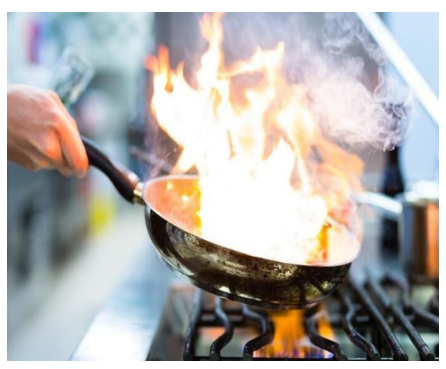

Use occupational protection

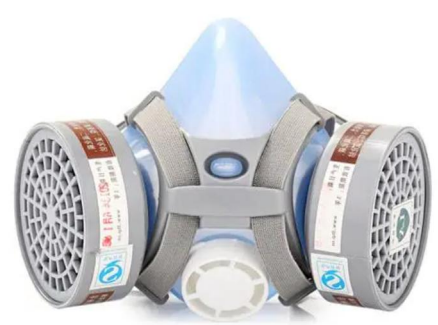

Exercise appropriately

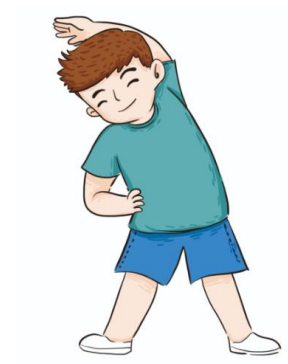

Avoid environmental pollution

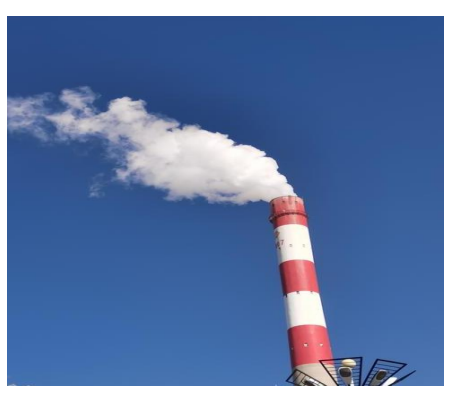

Eat fresh fruit and vegetables

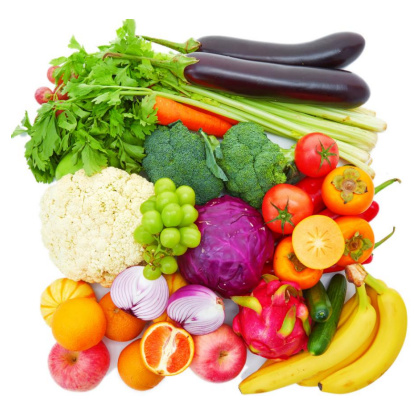

Participate in recommended screening

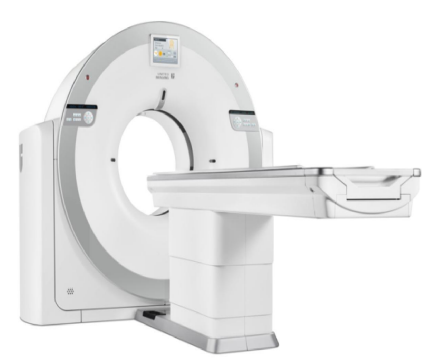

4. Do my family members need lung cancer screening?

Based on our previous research and relevant expert consensus, low-dose spiral CT screening for lung cancer is recommended for people aged 45–74 years who meet at least one of the following criteria:

- Smoking: at least 20 pack-years, including former smokers with at least 20 pack-years who quit less than 15 years ago;
- Passive smoking: living or working in the same room with a smoker for at least 20 years;
- Chronic obstructive pulmonary disease (COPD);
- Occupational exposure for at least 1 year to asbestos, radon, beryllium, chromium, cadmium, nickel, silica, soot, or coal dust;
- A parent, child, brother, or sister diagnosed with lung cancer.

Note: Pack-years = packs smoked per day (20 cigarettes per pack) × years of smoking.
